# Effects of hallucinogenic drugs on the human heart

**DOI:** 10.3389/fphar.2024.1334218

**Published:** 2024-02-02

**Authors:** Joachim Neumann, Stefan Dhein, Uwe Kirchhefer, Britt Hofmann, Ulrich Gergs

**Affiliations:** ^1^ Institut für Pharmakologie und Toxikologie, Medizinische Fakultät, Martin-Luther-Universität Halle-Wittenberg, Halle, Germany; ^2^ Rudolf-Boehm Institut für Pharmakologie und Toxikologie, Universität Leipzig, Leipzig, Germany; ^3^ Institut für Pharmakologie und Toxikologie, Medizinische Fakultät, Universität Münster, Münster, Germany; ^4^ Cardiac Surgery, Medizinische Fakultät, Martin-Luther-Universität Halle-Wittenberg, Halle, Germany

**Keywords:** bufotenin, psilocin, psilocybin, LSD, ergotamine, ergometrine, N,N-dimethyltryptamine

## Abstract

Hallucinogenic drugs are used because they have effects on the central nervous system. Their hallucinogenic effects probably occur via stimulation of serotonin receptors, namely, 5-HT_2A_-serotonin receptors in the brain. However, a close study reveals that they also act on the heart, possibly increasing the force of contraction and beating rate and may lead to arrhythmias. Here, we will review the inotropic and chronotropic actions of bufotenin, psilocin, psilocybin, lysergic acid diethylamide (LSD), ergotamine, ergometrine, N,N-dimethyltryptamine, and 5-methoxy-N,N-dimethyltryptamine in the human heart.

## Introduction

**FIGURE 1:**
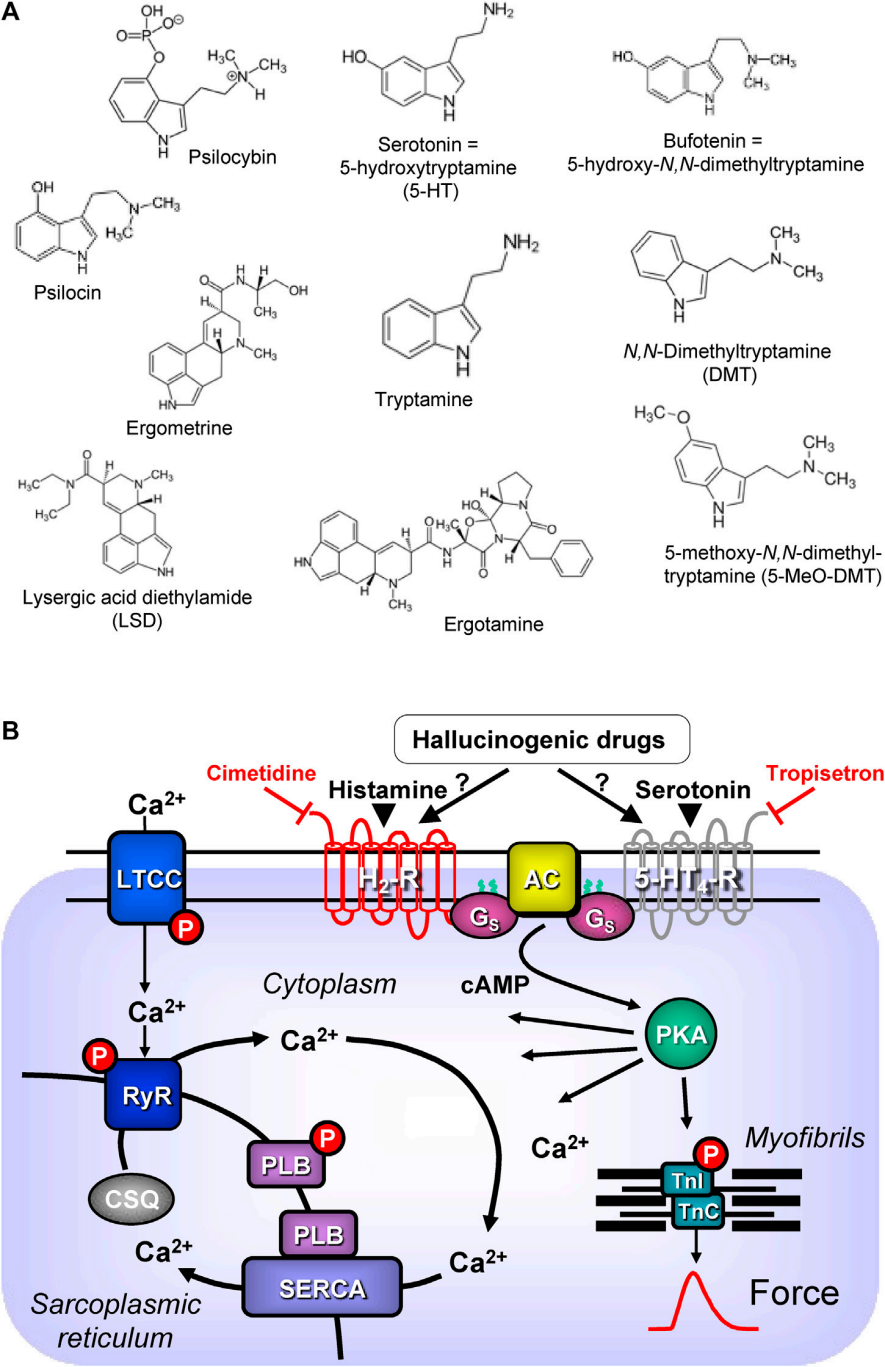
**(A)** Structural formulae of tryptamine derived hallucinogenic compounds. **(B)** Schematic drawing of the proposed signalling of hallucinogenic compounds in cardiac myocytes. Ca^2+^ enters the mammalian heart cell via the L-type Ca^2+^ channel (LTCC). This process can be enhanced by hallucinogenic compounds via a cascade starting in the sarcolemma via stimulation of Gs-protein (G_s_)-coupled 5-HT_4_ serotonin or H_2_ histamine receptors. Activation of adenylyl cyclase (AC) elevates subsequent production of cAMP and thereby activates cAMP-dependent protein kinase (PKA). PKA increases cardiac force generation and relaxation by increasing the phosphorylation state (P) of the L-type calcium channel (LTCC), of phospholamban (PLB) and of the inhibitory subunit of troponin (TnI). Trigger Ca^2+^ initiates release of Ca^2+^ from the sarcoplasmic reticulum via ryanodine receptors (RYR) into the cytosol. There, Ca^2+^ activates myofilaments and this activation leads to increased inotropy. In diastole, Ca^2+^ is taken up into the sarcoplasmic reticulum via a sarcoplasmic reticulum Ca^2+^-ATPase (SERCA), the activity of which is enhanced due to an increased phosphorylation state of PLB.

In this review, “drugs of interest” include the following organic molecules: bufotenin, psilocin, psilocybin, lysergic acid diethylamide (LSD), ergotamine, ergometrine, N,N-dimethyl-tryptamine and 5-methoxy-N,N-dimethyltryptamine. These drugs of interest (Figure 1A) are referred to as tryptamine derivatives. These drugs of interest are thus structurally similar to 5-hydroxyl-tryptamine (serotonin, 5-HT), the physiological agonist at serotonin receptors. Unlike indirect sympathomimetic drugs (e.g., metamphetamine, amphetamine), these compounds probably do not act solely or mainly as releasers of noradrenaline from storage sites in the human heart (Neumann et al., 2023a; Neumann et al., 2023b). In contrast, they are directly activate serotonin receptors in the heart (e.g., Jacob et al., 2023a). However, at least *in vitro* these tryptamines or related tiophene analogs may also act as monoamine transport releasers (Blough et al., 2014; Rudin et al., 2022). The hallucinogenic effects of these compounds are explained by the stimulation of 5-HT_2A_-serotonin receptors in the brain. In the heart, these drugs of interest can activate serotonin receptors. However, serotonin increases the force of contraction and beating rate in the human heart via 5-HT_4_-serotonin receptors and not via 5-HT_2A_-serotonin receptor (Neumann et al., 2017; Neumann et al., 2023a). In contrast to other species 5-HT_2A_- (rat) or 5-HT_3_-(guinea pig) serotonin receptors do not increase force in the human heart (Kaumann et al., 1990, reviews; Neumann et al., 2017; Neumann et al., 2023a). In order to provide a small animal model for human 5-HT_4_ serotonin receptors in the heart, we have generated transgenic mice that overexpress the human 5-HT_4_-serotonin receptor in the heart (5-HT_4_-TG). In cardiac preparations from 5-HT_4_ TG, serotonin increased the force of contraction (Gergs et al., 2010). Serotonin does not increase the force of contraction in isolated mouse cardiac preparations from wild-type mice (Gergs et al., 2010).

Interestingly, some of these drugs of interest (e.g., LSD) also activate histamine receptors, namely, H_2_-histamine receptors in the human heart. In the human heart, unlike in some animal hearts, H_2_-histamine receptors primarily mediate the positive inotropic or positive chronotropic effects of exogenous or endogenous histamine (reviews: Neumann et al., 2021a; Neumann et al., 2022; Neumann et al., 2023d). To study human H_2_-histamine receptors in a small animal model, we generated transgenic mice that overexpress the H_2_-histamine receptors in the heart (H_2_-TG), wherein histamine increased the force of contraction (Gergs et al., 2020; Neumann et al., 2021b; Neumann et al. 2021c; Neumann et al. 2021d; Neumann et al. 2021e; Gergs et al., 2021). Similar to serotonin, histamine does not increase the force of contraction in isolated cardiac preparations from wild type mice (Gergs et al., 2019).

5-HT_4_-serotonin and H_2_-histamine receptors share a common signal transduction system (Figure 1B). Both receptors are located on the outside of sarcolemma in cardiomyocytes and they couple to stimulatory G-proteins. Thereby they increase the activity of the adenylyl cyclases in the sarcolemma. Finally, both receptors lead to increased production of 3′, 5′-cyclic adenosine monophosphate (cAMP). This cAMP activates cAMP-dependent protein kinases in the cytosol of the cardiomyocytes. The cAMP is eventually degraded and inactivated by the action of phosphodiesterases. After stimulation of 5-HT_4_-serotonin and H_2_-histamine receptors, several target proteins in many compartments of the cardiomyocyte are phosphorylated and usually activated. A key role is played by the phosphorylation of the L-type Ca^2+^ channel (LTCC) in the sarcolemma. This leads to increased entering of trigger Ca^2+^ into the cardiomyocytes. This trigger Ca^2+^ then releases Ca^2+^ from intracellular stores in the sarcoplasmic reticulum (SR) and this Ca^2+^ activates the myofilaments. At the same time phosphorylation of phospholamban in the SR comes about. This mechanism increases the uptake rate of Ca^2+^ into the SR and this enhances relaxation of the heart muscle but also leads to higher filling of Ca^2+^ into the SR (Figure 1B). Thus, the next heart beat can be more vigorous because more Ca^2+^ can be released by trigger Ca^2+^ from the SR (Neumann et al., 2017; Neumann et al., 2022; Neumann et al., 2023c).

Except for LSD, all the drugs of interest occur naturally (Table 1). They are found mainly in plants or moulds. Some hallucinogenic compounds are present in high concentrations in animals, such as frogs or even in humans. The present review of the effects of the drugs of interest is limited to the mammalian heart, more specifically the human heart.

**TABLE 1:** Sources of the hallucinogenic drug.

	Source	References
Bufotenin	^1^Toad skin	^1^ Handovsky (1920)
	^2^Brosimum acutifolium	^2^ Moretti et al. (2006)
	^3^Anandenanthera peregrina	^3^ Ott (2001)
	^4^Human body	^4^ Forsström et al. (2001)
Ergometrine	Fungi: Claviceps purpurea	Dudley and Moir (1935)
Ergotamine	Fungi: Claviceps purpurea	Stange et al. (1998)
LSD: Lysergic acid diethylamide	Chemical laboratory	Hofmann (1959)
DMT: N,N-dimethyl-tryptamine	^1^Diplopterys cabreana	^1,2^ McKenna et al. (1984a)
	^2^Banisteriopsis caapi	^3,4^ Brito-da Costa et al. (2020)
	^3^Psychotria viridis	
	^4^Human brain	
5-methoxy-N,N-dimethyltryptamine	^1^Toad skin	^1^ Araujo et al. (2015)
	^2^Banisteriopsis caapi	^2^ McKenna et al. (1984b)
Psilocin	Fungi: psilocybe	Hofmann (1958), Hofmann (1959)
Psilocybin	Fungi: psilocybe	Hofmann (1958), Hofmann (1959)

The clinical use of this review will facilitate the safe usage of the drugs of interest. This knowledge is essential because nearly all drugs of interest have the potential to treat psychiatric diseases. In addition, during “recreational use”, overdoses of hallucinogenic drugs can occur. Then, it is helpful to have guidance on what antidotes might make sense from a pharmacological point of view.

### Bufotenin

Exogenous or endogenous serotonin (5-hydroxytryptamine, 5-HT) induces a positive inotropic effect, a relaxant effect, a positive dromotropic effect, and a positive chronotropic effect in the human heart via human 5-HT_4_-serotonin receptors (for reviews: Kaumann and Levy, 2006; Neumann et al., 2017; Neumann et al., 2023a). Studies on isolated porcine heart preparations have found that 5-HT can increase force and frequency via porcine 5-HT_4_-serotonin receptors (Kaumann, 1990; Villalón et al., 1990). In humans and porcine but not in other mammalian hearts like mice, cats, rats, dogs, or rabbits, 5-HT can augment force and beating rate via 5-HT_4_-serotonin receptors (Kaumann and Levy, 2006; Neumann et al., 2017; Neumann et al., 2023c; Neumann et al., 2023d).

Bufotenin (5-hydroxy-dimethyltryptamine) is structurally related to serotonin; it is a dimethylated (on the primary amine atom) form of serotonin (Figure 1). Hence, it is not surprising that, based on this similarity, bufotenin can bind to serotonin receptors and activate them. Indeed, *in vitro* bufotenin binds to 5-HT_2A_- and 5-HT_2C_- serotonin receptors (Almaula et al., 1996). Agonist binding to 5-HT_2A_-serotonin receptors might explain the hallucinogenic effects of bufotenin (Titeler et al., 1988). Moreover, bufotenin binds potently to 5-HT_1A_-, 5-HT_1B_- 5-HT_1D_-serotonin receptors (Dumuis et al., 1988).

In this context of affinities to various serotonin receptors, it seems necessary to discuss the possible detrimental effects of 5-HT_2B_- serotonin receptor stimulation for the heart. There is convincing evidence from cell culture work, animal studies, clinical retrospective and case control studies that in principle stimulation of 5-HT_2B_- serotonin receptor can induce proliferation of fibroblasts in the mammalian heart. This proliferation leads to abnormal thickening of leaflets of valves and can take place. This thickening can occur in the mitral leaflets, in tricuspid leaflets or on aortic cusps (Cosyns et al., 2013). This alteration in the anatomy of valves in the human heart can induce mitral insufficiency, tricuspid insufficiency or aortic insufficiency. This drug-induced valvular thickening is diagnosed by exclusion of other underlying pathologies (e.g., genetic defects or infections) and anamnesis of drug treatment by using echocardiography. Such alterations of the mitral valve and/or the aortic valve in the left heart are a burden to cardiac function and can lead to congestive heart failure. Similar damage to the tricuspid valves in the right heart will lead to pulmonary hypertension, like left ventricular heart failure a potentially deadly disease. In principle, any drug that stimulates 5-HT_2B_ -serotonin receptors can have such deadly consequences by the pathological pathway just mentioned because the 5-HT_2B_- serotonin receptor in the leaflets can lead to proliferation of local fibroblasts. Hence, bufotenin might damage the function of cardiac valves. On the other hand, stimulation of 5-HT_2B_- serotonin receptor probably has to be present for a prolonged period of time and with a sufficiently high occupancy of the 5-HT_2B_- serotonin receptor. Hence, if it were sufficient to treat patients for a short period of (e.g., every 3 months) with small doses of hallucinogenic drugs like bufotenine (smaller than 100 mg per os or 10 mg parenterally: Ott, 2001), then the harm for the cardiac valves could be acceptable (discussed in: McIntyre, 2023).

In this context, one should also mention effects of 5-HT on human coronary arteries, and human pulmonary arteries, because they may complicate therapy with hallucinogenic drugs. In brief, there is convincing evidence that serotonin can lead to vasoconstriction in coronary vessels (McFadden et al., 1991). This can lead to or at least may worsen ischemic heart disease because constriction of coronary arteries. This vasocontraction can occur via stimulation of 5-HT_2A_-serotonin receptors (Kaumann et al., 1994; Nilsson et al., 1999) and/or 5-HT_1B_-serotonin receptors (van den Broek et al., 2002) will lead to less perfusion of the heart. Likewise, pulmonary hypertension can be caused or aggravated if drugs stimulate 5-HT_2A_- or 5-HT_1B_ serotonin receptors in human pulmonary arteries (Cortijo et al., 1997). Indeed, bufotenin and most hallucinogenic drugs can activate to 5-HT_2A_-serotonin receptors and/or 5-HT_1B_-serotonin receptors (Dumuis et al., 1988; Almaula et al., 1996) and thus they may cause vasoconstriction. Whether this vasoconstriction occurs with bufotenin in humans is unclear and might be worth further studies.

Bufotenin exerted positive chronotropic effects in isolated spontaneously beating right atrial preparations from pigs, mediated by porcine 5-HT_4_-serotonin receptors (Medhurst and Kaumann, 1993). As far as we could find out, there are in the literature no binding data of bufotenin to 5-HT_4_-serotonin receptors. An interaction of bufotenin to 5-HT_4_-serotonin receptors is likely from the following experiments: bufotenin increased force of contraction and beating rate only in isolated left or right atrial preparations, respectively, of transgenic mice where the human 5-HT_4_-receptor was overexpressed in the heart (5-HT_4_-TG, Neumann et al., 2023e; Table 4). These effects were antagonized by 5-HT_4_ serotonin receptor antagonists (Neumann et al., 2023e). Moreover, in isolated human right atrial strips, which were paced to induce contraction, bufotenin likewise increased force of contraction and these effects were antagonized in transgenic mice by 5-HT_4_ serotonin antagonists (Neumann et al., 2023e; Table 4).

Bufotenin was first isolated to purity in Prague from toad skin (in Latin, *Bufo* means toad, Handovsky, 1920). The correct structural formula (they called it “5-Oxy-indolyl-äthyl-dimethylamin”) was found later in Munich and confirmed by synthesis (Wieland et al., 1934; Hoshino and Shimodaira, 1935, review; Chilton et al., 1979).

Bufotenin occurs not only in animals like toads but also in plants. Shamans in French Guiana used latex from Brosimum acutifolium to obtain hallucinogenic mixtures containing bufotenin (Moretti et al., 2006). Interestingly, bufotenin has been found in toads and the human body (Forsström et al., 2001). It might be formed enzymatically using a methyltransferase from serotonin (Figure 1) in human neuronal cells (Kärkkäinen et al., 2005). Bufotenin may underlie the fairy tale of the *Frog Prince* by the Grimm brothers (Siegel and McDaniel, 1991). In the fairy tale, kissing frogs may have released bufotenin from the frog’s skin (probably a toad). This bufotenin may have entered the human brain and led to hallucinations. Under these conditions, one might have confused the frog with a prince (Siegel and McDaniel, 1991).

Recently, a novel indolethylamine-N-methyltransferase in the skin and parotid glands of some toad species has been cloned (Chen et al., 2023). This enzyme probably underlies the production of bufotenin in the skin of particular toad species (e.g., *Bufo marinus*, *Bufo Bufo*) that are known to be used as sources of bufotenin (Chen et al., 2023). This novel enzyme is absent in common frogs (Chen et al., 2023). In toads, biosynthesis starts with tryptophan, which is hydroxylated to 5-hydroxytryptophan and then decarboxylated, leading to serotonin. The primary amine in serotonin is first methylated to monomethylserotonin. This secondary amine is then methylated again to the tertiary amine N,N-dimethylserotonin (bufotenin, Chen et al., 2023).

In the first paper on pure bufotenin, bufotenin was studied for its cardiac effects. While bufotenin (at high doses) did not alter the force of contraction in the isolated frog heart, it reduced the heart rate (Handovsky, 1920). Intravenous injection of bufotenin in dogs, cats, or rabbits increased blood pressure, but shortly after the injection, the animals died (Handovsky, 1920). However, these data are questionable. As noted above, no functional cardiac 5-HT_4_ serotonin receptors were present in these animals (dog: Chiba, 1977, cat and rabbit; Trendelenburg, 1960). The increase in blood pressure is likely not due to an increase in cardiac output, but probably due to peripheral vasoconstriction following stimulation of vascular arterial smooth muscle 5-HT_2A_-serotonin receptors in these animals.

Moreover, bufotenin can raise the phosphorylation state of phospholamban (Neumann et al., 2023e). Increased phosphorylation of phospholamban (Tada et al., 1976) leads to reduced time to relaxation and an increased rate of tension relaxation in atrial and ventricular preparations from 5-HT_4_-TG mice. Phosphorylated phospholamban de-inhibits the activity of the Ca^2+^ pump (Figure 1B) in the sarcoplasmic reticulum, thus increasing the rate at which calcium cations are pumped from the cytosol into the sarcoplasmic reticulum; fewer calcium cations bind to the myofilaments, and myofilaments relax faster (Hamstra et al., 2020).

This cardiac effect of bufotenin might play a clinical role (Table 2). Bufotenin can be taken orally to induce hallucinogenic effects, but perorally, high doses of bufotenin must be given in humans, because bufotenin seems to undergo a strong first-pass effect. Indeed, much higher peroral doses (100 mg) of bufotenin than parenteral doses (10 mg) are needed in humans to bring about hallucinogenic effects (self-experimentation: Ott, 2001).

**TABLE 2:** Clinical studies and tested indications of the hallucinogenic drug.

	Clinical studies in “ClinicalTrials.gov”	Some tested indications for the drugs in “ClinicalTrials.gov”	Remarks	„Pubmed“ hits
Bufotenin	5 (as metabolite of 5-Me-DMT)	Pharmacokinetics, Depression, Autism	Studied in depression Uthaug et al. (2019)	634
Ergometrine	9	Postpartum hemorrhage		2,781
Ergotamine	0		migraine	13,369
LSD: Lysergic acid diethylamide	8	Pharmacokinetics, Cluster headache, Palliative care	Studied in alcoholism, depression Passie et al. (2008); Bogenschutz et al. (2013)	5,638
DMT: N,N-dimethyl-tryptamine	0		Religious ceremonies Brito-da Costa (2020)	42
5-methoxy-N,N-dimethyltryptamine	10	Pharmacokinetics, Depression	Religious ceremonies Brito-da Costa (2020)	578
Psilocin	1	Comparison with psilocybin	Depression: Ross et al. (2016)	275
Psilocybin	130	Pharmacokinetics, depression, terminal illness, concussion headache, migraine, phantom limb pain, treatment of cocaine addiction, alcoholism, smoke cessation, interaction with serotonin reuptake inhibitors in depression, anorexia nervosa, post traumatic stress, binge eating, Morbus Alzheimer, burn out	Depression: Ross et al. (2016)	1834

In humans, bufotenin can be found physiologically in plasma. One might ask whether this bufotenin is clinically relevant. Indeed, plasma levels of bufotenin were elevated in patients with autism and schizophrenia (Emanuele et al., 2010; Table 3). On the one hand, one might hypothesise that these high levels of bufotenin might explain some of the hallucinations accompanying psychiatric diseases. On the other hand, elevated levels of bufotenin may lead to tachycardia in untreated patients. If that were the case, one could reduce the bufotenin-induced tachycardia with 5-HT_4_-serotonin receptor antagonists such as tropisetron or piboserod.

**TABLE 3:** “Therapeutic” and toxic plasma concentrations of the hallucinogenic drug in humans.

	Therapeutic	Toxic	References
Bufotenin	8–16 nM	Active metabolite of 5-Me DMT	Emanuele et al. (2010) (endogenous concentrations)
Ergometrine	4 nM		de Groot et al. (1993)
Ergotamine	0.69 nM^1^	15 nM^2^	^1^ Sanders et al. (1986)
			^2^ Stange et al. (1998)
LSD: Lysergic acid diethylamide	^1^3–9 nM	^2^33 μM	^1^ Holze et al. (2020)
			^2^ Mardal et al. (2017)
DMT: N,N-dimethyl-tryptamine	^1^0.38 µM		^1^ Strassman et al. (1994)
	^2^0.3 nM		^2^ Good et al. (2023)
5-Me DMT: 5-methoxy N,N-dimethyltrypta-mine	4 nM		Reckweg et al. (2021)
Psilocin	1 0.1 µM	^2^0.15 µM	^1^ Madsen et al. (2019)
			^2^ Lim et al. (2012)

Bufotenin has some beneficial effects on depressive patients (Uthaug et al., 2019). However, there is currently no accepted clinical indication for bufotenin. Over several decades, bufotenin and frog skins or plants containing bufotenin have sometimes been used as “recreational drugs” and have led to intoxication (Chamakura, 1994; Shen et al., 2010; Davis et al., 2018).

Bufotenin is an important active metabolite of the hallucinogenic compound 5-methoxy N,N-dimethyltryptamine (found in plants, *vide infra*). Bufotenin might be formed by metabolism in humans taking 5-methoxy N,N-dimethyltryptamine (Shen et al., 2010). One could treat severely ill patients with tropisetron, typically regarded as a 5-HT_3_-serotonin receptor antagonist. However, the tropisetron also blocks human 5-HT_4_-serotonin receptors (Kaumann et al., 1990) and is approved for use in humans in many countries. Alternatively, one can use the specific 5-HT_4_-serotonin receptor antagonist piboserod (Kjekshus et al., 2009), which has been used in at least one heart failure study in humans; thus, it might be used off-label, should the need arise in the patient.

### Lysergic acid diethylamide

Lysergic acid diethylamide (in the original publications in German: Lysergsäurediäthylamid: thence LSD) (LSD, Figure 1A) is an ergot derivative developed as an analeptic agent (review: Nichols, 2018). However, LSD turned out to be a hallucinogenic drug when Albert Hoffmann, the chemist at the Sandoz pharmaceutical company in Basel, Switzerland, who had synthesised LSD in 1938 AD, inadvertently ingested around 10–30 µg of LSD in 1943 (review: Nichols, 2018). At that time, LSD was the most potent hallucinogenic drug. LSD was first published in a scientific journal in 1947 (Nichols, 2018). Sandoz produced and gave LSD out to psychiatrists in Europe and the United States of America to look for potential clinical applications (Nichols, 2018). LSD (Delysid^®^) was studied in the 1960s in psychiatry with the hope of better understanding the molecular mechanisms of how psychosis is caused and to help with a psychotherapeutic approach to the patient (Nichols, 2018). However, from that time on, LSD was primarily used in illicit ways and, therefore, was practically removed from the legitimate drug market worldwide (Nichols, 2018). Currently, there is renewed interest in psychiatry in studying LSD in some contexts. The hallucinogenic effects of LSD are thought to be caused by the activation of 5-HT_2A_-serotonin receptors in the brain (Preller et al., 2017; review; Liechti et al., 2017), as with the other drugs of interest in this review.

In ligand binding studies, LSD had the following rank or of potencies: 5-HT_1A_- >5-HT_2A_- >5-HT_2C_- >5-HT_2B_- serotonin receptors. This rank order should be a little bit more specified: by far the highest affinity was displayed by LSD to 5-H_1A_-serotonin receptors and also the affinity at 5-HT_2A_-serotonin receptors and 5-HT_2C_- serotonin receptors is in the nanomolar concentration range. In contrast, the affinity for the 5-HT_2B_- serotonin receptor is much lower with about 10 µM (Rickli et al., 2016). Recent data also noted that LSD has an affinity for 5-HT_4_-serotonin receptors and H_2_-histamine receptors (around 10 µM for these receptors: Lewis et al., 2023). From these binding data at 5-HT_2B_-serotonin receptors one would assume that LSD can activate this receptor in the patient. This might lead valvular heart disease (*vide supra*). However, others claimed that any proofs for valvular damage through LSD from clinical studies is currently lacking (Tagen et al., 2023). However, this valvular side effect should be looked for in prospective clinical trials.

In isolated cardiac preparations, LSD was found to be a partial agonist at cardiac H_2_-histamine receptors in rabbit and guinea pig cardiac preparations (Angus and Black, 1980; Table 4). This conclusion was based on the following findings: LSD at low concentrations increased and at high concentrations reduced the beating rate in isolated right atrial preparations from rabbits in a cimetidine (a H_2_-histamine receptor antagonist)-sensitive fashion (Angus and Black, 1980). Moreover, LSD antagonised the positive inotropic effect of histamine in isolated guinea pig papillary muscles (Angus and Black, 1980). Currently, LSD is used primarily for “recreational” and “personal” purposes (Araújo et al., 2015), while some medical studies on its use in the treatment of alcoholism and depression are on record (Bogenschutz, 2003; Passie et al., 2008). Also, in Basel, Switzerland, from 2021 to 2023, a trial was recruited to test LSD versus placebo for the treatment of cluster headache pain (ClinicalTrials.gov Identifier: NCT03781128, Table 2).

**TABLE 4:** Cardiac effects in animal and human cardiac preparations of the drugs of interest.

	Animal studies	Human studies	References
Bufotenin	Positive chronotropic effect in isolated porcine atrial preparations via 5-HT_4_ receptors^1^, increase in force of contraction and in beating rate via 5-HT_4_ receptors in pigs^2^ and 5-HT_4_-TG^3^	Increase in force of contraction in isolated human right atrial preparations via 5-HT_4_ receptors^3^	^1^ Medhurst and Kaumann (1993)
			^2^ Villalon et al. (1990)
			^3^ Neumann et al. (2023e)
Ergometrine	Increase in left ventricular force of contraction in isolated perfused guinea pig heart via H_2_ receptors^1^, increase in force of contraction and beating rate in atrial preparations only via H_2_ receptors (H_2_-TG)^2^	Increase in force of contraction in isolated human right atrial preparations only via H_2_ receptors^2^	^1^ Bongrani et al. (1979)
			^2^ Jacob et al. (2023a)
Ergotamine	Increase in force of contraction and beating rate in atrial preparations via both H_2_- and 5-HT_4_-receptors (H_2_-TG, 5-HT_4_-TG)	Increase in force of contraction in isolated human right atrial preparations only via H_2_-receptors	Jacob et al. (2023b)
LSD: Lysergic acid diethylamide	Increase in force of contraction in guinea pig and rabbit ventricular preparations via H_2_ receptors^1^, increase in force of contraction and in beating rate in atrial preparations via both H_2_- and 5-HT_4_-receptors (H_2_-TG, 5-HT_4_-TG)^2^	Increase in force of contraction in isolated human right atrial preparations via both 5-HT_4_-receptors and H_2_ receptors^2^	^1^ Angus and Black (1980)
			^2^ Gergs et al. (2023)
DMT: N,N-dimethyl-tryptamine	Increase in force of contraction and beating rate in atrial preparations via 5-HT_4_ receptors (5-HT_4_-TG)	Increase in force of contraction in isolated human right atrial preparations via 5-HT_4_ receptors	Dietrich et al. (2023)
5-methoxy-N,N-dimethyltryptamine	Positive chronotropic effect in isolated porcine right atrial preparations via 5-HT_4_ receptors^1^, increase in force of contraction and beating rate in atrial preparations via 5-HT_4_ receptors (5-HT_4_-TG)^2^	Increase in force of contraction in isolated human right atrial preparations via 5-HT_4_-receptors^2^	^1^ Medhurst and Kaumann (1993)
			^2^ Dietrich et al. (2023)
Psilocin	Increase in force of contraction and beating rate in atrial preparations via 5-HT_4_ receptors (5-HT_4_-TG)	Increase in force of contraction in isolated human right atrial preparations via 5-HT_4_ receptors	Dimov et al. (2023)
Psilocybin	Increase in force of contraction and beating rate in atrial preparations via 5-HT_4_ receptors (5-HT_4_-TG)	Increase in force of contraction in isolated human right atrial preparations via 5-HT_4_ receptors	Dimov et al. (2023)

Low doses of LSD, given through the mouth in a solution of 0.5 mL volume (up to 26 µg) in healthy volunteers (male and female) led to a significant increase in systolic blood pressure, but not in heart rate or diastolic blood pressure. The missing effect of LSD in diastolic blood pressure and heart rate (mean values were higher) could be due to the low dosage of LSD. Indeed, in another study with more LSD, heart rate and diastolic blood pressure was found to be elevated: In this clinical study 200 µg LSD, given as an oral solution, increased systolic and diastolic blood pressure and heart rate in healthy subjects (male and female). These effects peaked at about 1 hour after drug application and returned to initial values within about 12 h (Holze et al., 2022). Under these conditions the peak plasma concentration of LSD was given as 25 ng/mL (Holze et al., 2022). In another clinical study from Switzerland, 100 µg of LSD was taken orally, there was an increase in body temperature, blood pressure, and heart rate compared to a placebo (Holze et al., 2020). In these probands, peak plasma concentrations of LSD ranged between 0.99 and 2.9 ng/mL (3.06–8.9 nM: Holze et al., 2020). In another study, the proportionality of plasma concentrations and doses taken per os for LSD was reported; a plasma half-life of 2.6 h for LSD and a first-order elimination pharmacokinetic behaviour of LSD were detected (Dolder et al., 2017). The use of nuclear magnetic imaging in the brain has deepened our understanding of the molecular actions of LSD in the human brain (Kaelen et al., 2016). Evidence for the binding of LSD to 5-HT_2A_-serotonin receptors may result this work (Kaelen et al., 2016).

At the time of this review, 122 studies of LSD had started, were ongoing or were going to be started (clinical.trials.gov, Table 2). In some of these studies, LSD was tested for the treatment of cluster headaches or depression. Hence, it might be of clinical interest that LSD can stimulate human H_2_-histamine receptors in the heart. A resultant tachycardia would be detrimental, especially by reducing the oxygen supply to the heart. These effects are even more overt in the presence of phosphodiesterase (PDE) inhibitors. In everyday life, PDEs can be inhibited by theophylline (in tea) or caffeine (in coffee beverages or power drinks). In patients, PDEs are inhibited when taking milrinone or levosimendan for heart failure or rolipram for asthma treatment. In such patients, special caution with LSD is warranted. One would recommend H_2_-histamine receptors and 5-HT_4_-serotonin receptor antagonists to treat tachycardia. Conceivably, prophylactic treatment, at least in patients suffering from angina pectoris with cimetidine, is indicated. This would not block the potential therapeutic agonist action of LSD on 5-HT_2A_ serotonin receptors or other serotonin receptors in the brain.

Intoxications with LSD are still being recorded (Liakoni et al., 2015; Li et al., 2019). In one series, the highest plasma concentration of LSD during intoxication amounted to 5.9 nM (McCarron et al., 1990). Brain tissue concentrations of up to 33 µM LSD (and metabolites) have been reported (Mardal et al., 2017), which are well in the range of the concentrations needed to elicit contractile effects in isolated cardiac preparations from H_2_-TG or the isolated human atrium (Gergs et al., 2023). Cardiovascular alterations during LSD intoxication include sinus tachycardia and hypertension (Blaho et al., 1997). One can probably recommend that the treatment of LSD intoxication should include an intravenously applied H_2_-histamine receptor antagonist, such as cimetidine or ranitidine.

LSD binds to many receptors (e.g., several isoforms 5-HT-receptors) (Roth et al., 2002). Notably, LSD binds as an agonist to 5-HT_2A_- and 5-HT_2B_- serotonin receptors and the crystal structure of LSD bound to 5-HT_2B_- serotonin receptors is now known (Wacker et al., 2017). LSD led to tachycardia in users (e.g., Holze et al., 2020). Indeed, we noted contractile effects in atrial and ventricular preparations of LSD in H_2_-TG and 5-HT_4_-TG (Gergs et al., 2023). In isolated human right atrial preparations, LSD increased the force of contraction via H_2_- and 5-HT_4_-serotonin receptors (Gergs et al., 2023). However, it is currently not known whether LSD increases ventricular function in the human heart. This is an interesting question to study. In the ventricles of humans, H_2_-histamine receptors are present and functional in failing human hearts (Bristow et al., 1982; Baumann et al., 1983; Matsuda et al., 2004). 5-HT_4_ serotonin receptors are likewise expressed in the human ventricle. However, 5-HT increased force only in isolated failing human ventricles, but not in isolated non-failing ventricles (review: Neumann et al., 2023c). In non-failing ventricular human preparations, serotonin only increased the force of contraction when initially a phosphodiesterase inhibitor was given (Neumann et al., 2023c; Table 4).

Hence, it is likely that LSD stimulates force in the ventricle, but this remains a hypothesis. In the absence of a PDE inhibitor, LSD concentration dependently reduced the force of contraction (Jacob et al., 2024). These effects may be due to the antiadrenergic effects of LSD. Indeed, early binding data have reported an affinity of LSD to β-adrenergic receptors (Dolphin et al., 1978). It was noted that after pretreatment with the β-adrenoceptor agonist isoprenaline, LSD concentration dependently reduced the force of contraction in the isolated human atrium (Jacob et al., 2024). Similarly, Angus and Black (1980) found that in guinea pig papillary muscles, LSD antagonised the positive inotropic effects of histamine. Likewise LSD inhibited cAMP formation that was stimulated by histamine (Green et al., 1977). Consistent with the general concept that LSD is a partial agonist at serotonin receptors, after prestimulation with serotonin, LSD exerts a concentration-dependent negative inotropic effect in human right atrial preparations (Jacob et al., 2024). In summary, LSD behaves as a partial agonist in histamine and serotonin receptors and as an antagonist at β-adrenergic receptors in the human isolated atrium. The clinical consequences of this warrant further investigation.

### Ergotamine

Ergotamine and LSD share the lysergic acid moiety (Figure 1A). Hence, it may not be surprising that ergotamine, like LSD, can bind to 5-HT_2A_-serotonin receptors in the brain. As with LSD, ergotamine can lead to hallucinations (Gulbranson et al., 2002; Silberstein and McCrory, 2003). Ergotamine can also stimulate peripheral 5-HT_2A_-serotonin receptors but also, as a partial agonist, vasoconstrictory α_1_-adrenoceptors (review: Silberstein and McCrory, 2003). Ergotamine is found in fungi like Claviceps purpurea that grow on cereals and still causes arterial constrictions, but possibly also hallucinations in consumers of cereals (e.g., Stange et al., 1998; Liegl and McGrath, 2016; Cervellin et al., 2020; Huybrechts et al., 2021). Moreover, ergotamine is also degraded by the cytochrome CYP2D6; some cases of ergotamine intoxication have been reported when patients are additionally treated with drugs that are inhibitors of CYP2D6 (Mohamedi et al., 2021).

Ergotamine is also binding to 5-HT_2B_- serotonin receptors (Fitzgerald et al., 2000). This binding to and activation of 5-HT_2B_- serotonin receptors may explain why ergotamine was the first drug reported to lead to valvular heart disease (review: Ledwos et al., 2022). One has argued the ergotamine was given in these cases continuously over a long time, e.g., to migraine patients. This long lasting stimulation of 5-HT_2B_-serotonin receptors for the reasons discussed above (section on bufotenin) may explain these detrimental effects of ergotamine (Ledwos et al., 2022).

Ergotamine is formed in fungi from lysergic acid to which alanine, proline and phenylalanine are covalently linked (Jamieson et al., 2021). No inotropic effect of ergotamine was found in isolated paced cat papillary muscles (Rabinowitz et al., 1975). However, this is a species problem because H_2_-histamine receptors and 5-HT_4_-serotonin receptors are functionally absent in the cat heart (Laher and McNeill, 1980, review; Neumann et al., 2021a). In contrast, a close derivative of ergotamine, called ergometrine (Figure 1), has been shown to elicit an increase in force in the guinea pig heart, which contains functional H_2_-histamine receptors (review: Neumann et al., 2021a). In intoxications (Table 3), much high plasma levels of ergotamine, such as 0.015 µM, have been reported (Stange et al., 1998), which could be agonistic in cardiac preparations.

Interestingly, ergotamine was an agonist at the human H_2_- histamine and serotonin 5-HT_4_-receptors in the transgenic mouse atrium (Jacob et al., 2023b; Table 4). This is not without precedence. Ergotamine acts on many G-protein coupled receptors (Silberstein and McCrory, 2003). However, In isolated human right atrial preparations ergotamine increased force of contraction only via H_2_-histamine receptors (Jacob et al., 2023b). As with LSD, one noted with ergotamine alone a time- and concentration-dependent negative inotropic effect. This negative inotropic effect of ergotamine is not due to the blocking of β-adrenergic receptors (Jacob et al., 2024).

## Ergometrine (ergobasine, ergonovine and ergotocine)

Ergometrine is on the list of essential drugs of the World Health Organisation (WHO, 2021). Like ergotamine, ergometrine is closely related to LSD (Figure 1). In LSD, the primary lysergic acid molecule contains two diethyl substituents in the amino group of its amide derivative (Meneghetti et al., 2020). In the molecule of ergometrine, there is at this position only one substituent, namely, an isopropanolol group (lysergic acid beta-propanolamine: Stoll, 1936; Stoll and Burckhardt, 1935; Thomson, 1935).

As mentioned above, in the ergoline ring that is part of the lysergic acid structure, one can discern structural elements of at least four neurotransmitters: serotonin, dopamine, noradrenaline and histamine (Figure 1A). Hence, the agonistic or antagonistic action of ergometrine on the receptors of these four neurotransmitters can be predicted. These four neurotransmitters use more than one receptor. As a result, a broad spectrum of action via diverse receptors is expected with ergometrine and is indeed a clinical and experimental observation. Ergometrine can stimulate α_1_-and α_2_-adrenoceptors, leading to vasoconstriction in rats (Kalkman et al., 1982). Moreover, ergometrine stimulates 5-HT_1_-serotonin receptors, which can induce vasoconstriction (Bai et al., 2004). Ergometrine can also act as a partial agonist at 5-HT_2A_ serotonin receptors (Hollingsworth 1988. Stimulation of these HT_2A_ serotonin receptors in humans can lead to vasoconstriction (Kaumann et al., 1994; van den Broek et al., 2002). If resistance vessels in the periphery constrict, hypertension would follow. If vasoconstriction via HT_2A_ serotonin receptors occurs in the coronary arteries, angina pectoris can follow (Kaumann and Levy, 2006). Hence, several serotonin receptors alone or combined could explain why ergometrine can cause vasoconstriction.

Peripheral vasoconstriction due to ergometrine has probably been noted since the Middle Ages in Europe (review: Grzybowski et al., 2021). Ergometrine constricts the arteries of the legs, arms, and coronary arteries in susceptible patients. This detrimental effect is sometimes used for diagnostic purposes in cardiology. In some countries, ergometrine is given to intentionally induce contraction of the coronary arteries. In this way, patients with variant angina or “Prinzmetal angina” can be better diagnosed (Romagnoli et al., 2005; Koizumi et al., 2006; Sueda et al., 2017; Picard et al., 2019).

Interestingly, there are cases in which ergometrine has probably induced atrial fibrillation in postpartum women (Birch et al., 2019). These arrhythmias could be due to the stimulation of receptors, as ergometrine binds to and stimulates human H_2_-histamine and 5-HT_4_-serotonin receptors (Jacob et al., 2023a) and because H_2_-histamine and 5-HT_4_-serotonin receptors can cause cardiac arrhythmias (review: Neumann et al., 2021a; Neumann et al., 2023c).

As mentioned above, ergometrine is agonistic at 5-HT_2A_ serotonin receptors (Hollingsworth et al., 1988). This interaction in the brain may lead to hallucinations (animal studies: Balsara et al., 1986, humans; Ott and Neely, 1980). In patients, intoxication with ergometrine is rare. However, there are case reports that imply the misuse of ergometrine-containing plants. Seeds of the Hawaiian baby woodrose (argyreia nervosa) led to hallucinations in humans (Klinke et al., 2010).

Ergometrine stimulates H_2_-histamine receptors in guinea pig perfused hearts (Bongrani et al., 1979; Table 4). Moreover, ergometrine increased force of contraction and beating rate in left or right atrial preparations from H_2_-TG and from 5-HT_4_-TG via human H2-histamine receptors and 5-HT4-serotonin receptors (Jacob et al., 2023a). However, ergometrine was more effective via H_2_-histamine receptors than via 5-HT_4_-serotonin receptors (Jacob et al., 2023a). In addition, ergometrine via H_2_-histamine receptors can increase the force of contraction in isolated human right atrial preparations if a phosphodiesterase inhibitor is present but only via H_2_-histamine receptors and not via 5-HT_4_-serotonin receptors (Jacob et al., 2023a). Like ergotamine and LSD, ergometrine induced (in the absence of a phosphodiesterase inhibitor) a negative inotropic effect (Jacob al. 2023a).

Phosphodiesterases degrade cAMP and thus inactivate cAMP. The most relevant phosphodiesterase in the human heart is called phosphodiesterase III (Kamel et al., 2023). If this phosphodiesterase III is inhibited by milrinone or cilostamide, then the effect of cAMP producing pathways is amplified because less cAMP is inactivated and thus more cAMP is functional to lead to positive inotropic effects (Feldman et al., 1987). Thus, inhibition of phosphodiesterases is sometimes used to amplify receptor mediated positive inotropic effects in human cardiac preparations.

We noted that this negative inotropic effect of ergometrine is similar to that of LSD and due to antagonistic action at β-adrenoceptors (Jacob et al., 2024). Moreover, normal therapeutic peak plasma concentrations of ergometrine (used in gynaecology) are 4 nM (Table 3) and are thus too low to affect contractile functions (Jacob et al., 2023a). In cases of intoxication with ergometrine or ergometrine-containing extracts, higher ergometrine concentrations might be active on the heart.

## N,N-dimethyltryptamine (DMT)

N,N-dimethyltryptamine is structurally related to serotonin (5-hydroxytryptamine) because it is a substituted tryptamine derivative with methyl moieties at the aliphatic amino group. Hence, it is not surprising that, based on this similarity to serotonin, N,N-dimethyltryptamine can bind to serotonin receptors. Agonist binding to 5-HT_2A_-serotonin receptors is thought to explain the hallucinogenic effects of N,N-dimethyltryptamine (Titeler et al., 1988). N,N-dimethyltryptamine exerted positive chronotropic effects in isolated spontaneously beating hearts from rabbits (Fozard and Ali, 1978). However, the contractile effects of 5-HT in rabbit atria are not mediated by 5-HT_4_-serotonin receptors but by the release of noradrenaline (Trendelenburg, 1960). Hence, the effects of DMT in rabbit hearts were not 5-HT_4_-serotonin receptor-mediated.

N,N-dimethyltryptamine occurs in many plants (Rätsch, 2015) and is used as a recreational psychedelic drug (global prevalence studied by Winstock et al., 2014) and even for ritual or religious purposes (McKenna et al., 1984a, review; Gable, 2007). DMT was found in the leaves of the plant Diplopterys cabrerana in Ecuador and Colombia (Ott, 1999; Brito-da Costa et al., 2020). However, DMT is also synthesised in the human brain and may be a neurotransmitter in humans (review: Carbonaro and Gatch, 2016). DMT was initially synthesised out of sheer chemical curiosity without studying biological responses in humans (Manske, 1931). In some species of toads, DMT was also detected. As in other animals, tryptophan is decarboxylated to tryptamine in toads. The decisive next step is catalysed by the high turnover rates of a particular enzyme in some species of toads (as mentioned above for bufotenin). Tryptamine is then sequentially methylated via monomethyltryptamine to DMT via a newly cloned indolethylamine methylase found, especially in *Bufo marinus* (Chen et al., 2023).

The leaves of the *Psychotria viridis* bush contain DMT. The bark of a plant (*Banisteriopsis caapi* vine) and contains harmala alkaloids which can inhibit the activity enzyme monoamine oxidase A (MAO-A) (Brito-da-Costa et al., 2020). This mixture, called ayahuasca, has been used since pre-Columbian times by indigenous tribes of the Amazon Basin (Gable, 2007). Ayahuasca is used for medical purposes (Brito-da-Costa et al., 2020). However, if extracts containing only DMT were drunk, the DMT would be rapidly inactivated by the MAO-A in the stomach lining. Therefore, users included plant extracts (here: harmala alkaloids) that contain MAO-A inhibitors (which at higher concentrations also inhibit monoamine oxidase B (MAO-B) (reviewed in: Callaway et al., 1999) when they used ayahuasca (McKenna et al., 1984a).

As with ayahuasca, pure DMT applied perorally alone does not lead to hallucinations due to the strong first-pass effect. DMT is metabolised in the gut and liver (McKenna et al., 1984a; Ott, 1999) like perorally applied serotonin. However, MAO activity (an example of a first-pass effect) of the gastrointestinal tract is anatomically avoided, such as when smoking or via injection of DMT or insufflation of DMT. In this case, DMT is active (Gable, 2007). Moreover, if the metabolism of DMT is impaired by drugs, hallucinogenic effects will occur.

In many countries, DMT use is restricted out of fear of misuse. One can argue that the beneficial effects of DMT, for instance, in psychiatric patients, might be considerable because the toxicity of DMT is low, and few deaths from DMT have been reported (Brito-da-Costa et al., 2020). The DMT content in *Psychotria viridis* bush and of β-carboline alkaloids in *Banisteriopsis caapi* vine ranges from 3–9.5 or 0.05%–1.95% mg/g dry weight, respectively, indicating high variability of doses taken and thus of pharmacological outcome (McKenna et al., 1984a; Callaway et al., 1996; Callaway et al., 1999; Callaway et al., 2005). Ayahuasca contains 0.14–0.6 mg/mL, equal to a total daily dose of 33–36 mg (Gable, 2007). As expected, injection of DMT leads to cognitive effects faster than taking ayahuasca (10 min versus 60 min), and the psychological effects are more potent due to a higher peak plasma concentration of DMT after injection of the same dose (Riba et al., 2015). Interestingly, some species of nutmeg, namely, Virola (Myristicaceae), contain high concentrations of DMT and at least minute amounts of MAO-inhibitory β-carbolines (McKenna et al., 1984b). A resin prepared from the bark of Virola is used by autochthonous Amazon tribes for hallucinogenic purposes (Plotkin and Schultes, 1990).

There is some debate as to the toxicity of DMT (Cameron et al., 2018) The lethal dose of DMT in mice is around 47 mg/kg if given intraperitoneally (Gable, 2007). Based on rodent studies, the dose where half of the patient would die (LD_50_) of DMT in men is estimated at 1.6 mg/kg given intravenously (Gable, 2007). There have not been recorded deaths due to ayahuasca, but when polypharmacy is used and pure 5-methoxy-DMT is added, at least one human death is found in the literature (Sklerov et al., 2005).

In ligand binding studies, DMT had the following rank or of potencies: 5-HT_1A_- >5-HT_2A_- > 5-HT_2C_- > 5-HT_2B_ (3.4 µM) serotonin receptors. The highest affinity was displayed by DMT to 5-H_1A_-serotonin receptors with 75 nM. The affinity for 5-HT_2C_-serotonin is much lower, about 420 nM (Rickli et al., 2016). DMT inhibited transporters with most potent inhibition for serotonin-transporter, then noradrenaline-transporter and lowest at dopamine-transporter (52 μM, Rickli et al., 2016). For adrenergic and dopaminergic receptors the rank order of affinity of DMT was: α_1_-adrenoceptor > α_2_-adrenoceptor > D_2_-dopamine receptor > D_1_-dopamine receptor (Rickli et al., 2016). From these binding data at 5-HT_2B_-serotonin receptors, one would assume that DMT can activate this 5-HT_2B_-serotonin receptor in the patient only under certain conditions. This might lead valvular heart disease (*vide supra*). However, others claimed that any proofs from clinical studies is currently lacking for valvular damage by DMT (Tagen et al., 2023). However, this side effect should be looked for in prospective clinical trials.

Initial studies of pure DMT administered intramuscularly in normal volunteers (0.7–1.1 mg/kg body weight) led to rapid (5–10 min) brief (1 h) visual hallucinations, euphoria, mydriasis, and an increase in blood pressure (Szára, 1956). In a placebo-controlled study in humans, intravenous application of 0.3 mg/kg DMT led to peak DMT plasma levels (at about 5 min after injection) of 70 ng/mL (about 0.38 μM, Table 3) and increased heart rate and blood pressure. Additional results included increased temperature, adrenocorticotropic hormone, prolactin, and cortisol levels in plasma (Strassman et al., 1994). Similarly, using ayahuasca preparations from the Amazon Basin in human volunteers, the half-life of DMT was reported as about 260 min, with a volume of distribution of about 55 L per kilogram. Temperature, heart rate, blood pressure, pupil diameter, and breathing rate increased (Callaway et al., 1999). The plasma concentration of harmine, another tryptamine derivative, and MAO inhibitor peaked when drunk with ayahuasca brew at about the same time as DMT, with a similar volume of distribution (Callaway et al., 1996; Callaway et al., 1999). These findings may mean that the plant contains not only the hallucinogenic compound but also some other related ingredient that improves the bioavailability of the hallucinogenic compound, at least in part. DMT binds to 5-HT_1A_, _1B_, _1D_, - and 5-HT_2A_, 5-HT_2B_, 5-HT_2C_, 5-HT_6_ - and 5-HT_7_ -serotonin receptors (Deliganis et al., 1991; Brito-da-Costa, 2020). Binding to 5-HT_4_ serotonin receptors has never been reported to the best of our knowledge.

### 5-methoxy-N,N,-dimethyltryptamine (5-Me-DMT)

5-methoxy-N,N-dimethyltryptamine is also structurally related to serotonin (5-hydroxytryptamine) because it is a substituted tryptamine derivative (Figure 1). The molecule 5-methoxy-N,N-dimethyltryptamine is found in plants and animals (Ott, 2001; Araújo et al., 2015). Perorally given alone, 5-methoxy-N,N-dimethyltryptamine is rapidly metabolised by monoamine oxidases in the gastrointestinal tract to inactive metabolites (Shen et al., 2010). Hence, it is used parenterally or in combination with inhibitors of the enzymatic activity of monoamine oxidases (Shen et al., 2010). These inhibitors could be antidepressant drugs, such as tranylcypromine. There are also reports in the literature that pure 5-methoxy-N,N-dimethyltryptamine was mixed with plant extracts containing the natural monoamino oxidase inhibitor harmaline, which eventually brought the user to the intensive care unit because he was intoxicated (Brush et al., 2004).

In anaesthetised rats, 5-methoxy-N,N-dimethyltryptamine reduced heart rate and blood pressure (Dabiré et al., 1987). These effects have been suggested to be due to the stimulation of 5-HT_1_ serotonin receptors (Dabiré et al., 1987). The interpretation of the data in rat might be made complicated because Dabiré et al., 1987 used anaesthesia during their experiments. The anaesthesia might have exerted powerful modulatory effects on cardiac responsiveness. In contrast, we reported that 5-HT increased the force of contraction in isolated rat hearts via 5-HT_2A_ serotonin receptors (Läer et al., 1998).

In pithed rats, 5-methoxy-N,N-dimethyltryptamine failed to affect the beating rate of the heart (Dabiré et al., 1992). Surprisingly, the rat heart contains inotropically functional 5-HT_2A_ serotonin receptors (Läer et al., 1998). The beating rate in narcotised rats or neonatal rat cardiomyocytes could be increased by serotonin (Higgins et al., 1981; Torres et al., 1996). In the isolated blood-perfused rat heart, minor positive chronotropic effects but significant inotropic effects of 5-HT were observed (Sakai and Akima, 1979). These divergent findings might result from methodological differences.

5-methoxy-N,N-dimethyltryptamine is found in plants and toads. It is often prepared from the Sonoran Desert toad (a toad with very high concentrations of 5-methoxy-N,N-dimethyltryptamine in the parotids and the skin) in the southern United States of America and Central and South America for ritual or recreational purposes (Araújo et al., 2015). 5-methoxy-N,N-dimethyltryptamine is a substrate for CYP2D6. CYP2D6 converts 5-methoxy-N,N-dimethyltryptamine to bufotenin (5-hydroxy-N,N-dimethyltryptamine, see above), also a naturally occurring (in toad skin or toad venom) hallucinogenic compound (review: Eichelbaum, 2003; Shen et al., 2010, *vide supra*). The expression of CYP2D6 is genetically regulated. Thus, slow and fast metabolisers are expected to experience longer or slower responses to 5-methoxy-N,N-dimethyltryptamine (review: Eichelbaum, 2003; Shen et al., 2010). Inhibitors of CYP2D6 are expected to prolong the hallucinogenic effects of 5-methoxy-N,N-dimethyltryptamine, but this has not yet been reported in patients.

One could speculate that the hallucinogenic effects of 5-methoxy-N,N-dimethyltryptamine result, at least in part, from bufotenine which is an active metabolite of 5-methoxy-N,N-dimethyltryptamine (Figure 1A). High concentrations of 5-methoxy-N,N-dimethyltryptamine are found in the bark and leaves of some species of the Virola plant (Myristicaceae, nutmeg) in the federal state Amazonas of the Union of Brazil (review: Ott, 2001). Extracts of the aforementioned plants were used as snuffs in shamanic ceremonies in South America dating back to pre-Columbian times (Ott, 2001). Preparations from species of Virola contained varying amounts of 5-methoxy-N,N-dimethyltryptamine (ranging from 0.017% to 1.57% of weight), sometimes together with smaller amounts of DMT. Hence, 5-methoxy-N,N-dimethyl-tryptamine is currently thought to be the main hallucinogenic principle of Virola extracts or Virola-containing pasts (Ott, 2001).

5-methoxy-N,N-dimethyltryptamine is psychoactive in various routes of application: 5-methoxy-N,N-dimethyltryptamine can be injected intravenously, can be breathed as a vapour, used as a snuff or as an errhine. In addition 5-methoxy-N,N-dimethyltryptamine can be given intranasally or sublingually, but also perorally in humans (Ott, 2001; Table 3). Typically, 10 mg (0.14 mg(kg) of chemical pure 5-methoxy-N,N,-dimethyltryptamine induced (in all the galenic forms mentioned) a hallucinogenic effect in humans (self-experiments: Ott, 2001). The addition of MAO inhibitors (harmaline 3.7 mg and a higher free base) potentiated the hallucinogenic effect of 5-methoxy-N,N-dimethyltryptamine, at least when using them nasally, sublingually, and perorally in humans (self-experiments: Ott, 2001). On the other hand, this seems to imply that it is active on its own, regardless of the additional presence of an MAO inhibitor, in contrast to DMT. In the human heart, 5-methoxy-N,N-dimethyltryptamine is more potent and effective than DMT in raising the force of contraction, at least in isolated human atrial preparations (Dietrich et al., 2023; Table 4).

Recreational drugs like N,N-dimethyltryptamine and 5-methoxy-N,N-dimethyltryptamine have led to intoxications (Brush et al., 2004). Our data might argue that these intoxications can involve the heart and that cardiac side effects could be treated by 5-HT_4_ receptor antagonists (Dietrich et al., 2023; Table 4). From a practical point of view, one could treat severely ill patients with tropisetron. Our data indicate that tropisetron can reduce the cardiac effects of 5-methoxy-N,N-dimethyltryptamine on human 5-HT_4_ serotonin receptors. Currently, there are 14 studies of N,N-dimethyltryptamine and two of 5-methoxy-N,N-dimethyltryptamine (clinical trials.gov, Table 2). The main indication in these clinical trials was depression. 5-methoxy-N,N-dimethyltryptamine is metabolised by CYP2D6 (Shen et al., 2010). The potency of 5-methoxy-N,N-dimethyltryptamine to increase the force of contraction could be increased by pretreatment of human atrial preparations from 5-HT_4_-TG in combination with the phosphodiesterase inhibitor cilostamide (Dietrich et al., 2023). As already mentioned above, In everyday life, PDEs can be inhibited by theophylline (in tea) or caffeine (in coffee beverages or power drinks). In patients, PDEs are inhibited when taking milrinone or levosimendan for heart failure or rolipram for asthma treatment. In such patients, special caution is warranted with 5-methoxy-N,N-dimethyltryptamine, based on our data (Dietrich et al., 2023; Table 4).

### Psilocin

Psilocin (Table 1A) is chemically related to serotonin (Hofmann et al., 1958; Hofmann et al., 1959). Psilocin and its precursor, psilocybin, can be described as substituted indole derivatives, namely, [3-(2-dimethylaminoethyl)-1H-indol-4-yl] dihydrogen phosphate and 4-hydroxy-N,N-dimethyltryptamine, respectively (Hofmann et al., 1958; Hofmann et al., 1959; Figure 1A). Psilocin has a high affinity to many receptors, mainly 5-HT_2A,B,C_ (pdsp.unc.edu., Halberstadt and Geyer, 2011), but its affinity to 5-HT_4_ serotonin receptors has not yet been reported (McKenna et al., 1990). The Food and Drug Administration (FDA) in the United States of America has since given psilocybin a fast-track designation for depression (Hesselgrave et al., 2021). Clinical studies have found that psilocybin might be useful in treating alcoholism, tobacco addiction, depression, and anxiety in cancer patients (discussed in Geiger et al., 2018).

In ligand binding studies, psilocin had the following rank or of potencies: 5-HT_2A_- >5-HT_2C_- >5-HT_1A_-serotonin-receptors (Rickli et al., 2016). It has been recently suggested that psilocybin might be chemically modified such that a derivate still acts as an antidepressant but is devoid of unwanted hallucinogenic effects which are currently thought to result from binding of psilocin to 5-HT_2A_-serotonin receptors (Hesselgrave et al., 2021). There was practically no affinity of psilocin for the 5-HT_2B_-serotonin receptor (larger than 20 μM, Rickli et al., 2016). From these binding data of psilocin at 5-HT_2B_-serotonin receptors, one would assume that psilocin cannot activate this receptor in the normal client or patient. Likewise, there is not any proof from clinical studies for valvular damage due to psilocin (Tagen et al., 2023). However, this side effect should be looked for in prospective clinical trials. The affinity of psilocin at others receptors probably does not play a clinical role. For instance, the affinity at the most sensitive adrenergic receptor, the α_2_-adrenoceptor amounts to 2.1 µM (Rickli et al., 2016). Likewise, psilocin probably does not act clinically via inhibition of the serotonin transporter (SERT) activity because its affinity for SERT is too low. For instance, a Ki value of 3.8 μM (Poulie et al., 2019) at SERT was reported. Such a high concentration is not reached with therapeutic dosage of psilocin (e.g., 0.1 µM plasma concentration of psilocin. Madsen et al., 2019).

Comparing the structural formulae of 5-HT and psilocin, it is obvious that psilocin is different in two regards: 1) psilocin contains hydroxyl-moiety at C4, not C5 of the indole ring, and 2) the amine function is doubly methylated (Figure 1A). Psilocybin is dephosphorylated to psilocin by alkaline phosphatases that occur in the blood and in many tissues (*in vitro* dephosphorylation of psilocybin: Horita and Weber, 1962; *in vivo* dephosphorylation of psilocybin in humans; Hasler et al., 1997). Psilocin is a structural isomer of bufotenin, chemically 5-hydroxy-N,N-dimethyltryptamine, and is hallucinogenic (*vide supra*, Figure 1A). Psilocybin is regarded as a prodrug, and the active metabolite formed in humans is psilocin (Hasler et al., 1997). Psilocybin and psilocin are found in many fungi from the genus Psilocybe (review: Nichols, 2020). The name was coined using ancient Greek, from the appearance of the fungi to botanists: psilos (ψιλος, naked) kube (κυβη, head) (Rätsch, 2015). These fungi have been used in religious ceremonies since prehistoric times in some parts of the world (Geiger et al., 2018). They have been called “magic mushrooms” because they can cause mind-altering experiences like hallucinations. The active ingredients of the fungi are, therefore, often classified as hallucinogenic drugs. The active ingredients were identified by Albert Hofmann, a Swiss organic chemist known as the inventor of LSD, in mushrooms from Central Mexico; he also synthesised psilocin and psilocybin *in vitro* (Hofmann et al., 1958; Hofmann et al., 1959).

These magic mushrooms and their ingredients are popular recreational drugs in the United States of America. Moreover, psilocybin was detected in several other fungi or moulds, namely, Conocybe spp. Galerina steglichii, Inocybe spp. and Pluteus spp. (Rätsch, 2015). Psilocybin is not produced in human cells, but more generally in mammals, conceivably because crucial synthetic enzymes are lacking in animals that are present in fungi. The synthesis of psilocybin in fungi and the enzymes involved its synthesis in fungi have been presented by others (Geiger et al., 2018). In brief, in fungi, psilocybin is formed from L-tryptophan, which is decarboxylated to tryptamine; the next steps are hydroxylation, phosphorylation, and methylation, ending with psilocybin (Fricke et al., 2017). Psilocin can be metabolised via side-chain oxidation and the formation of glucuronides, and it has a half-life of about 3 hours in humans (Geiger et al., 2018). The enzymes involved have not yet been clearly described. However, if they are the typical cytochromes described above, drugs that inhibit cytochromes are predicted to prolong the half-life and, thus, the pharmacological action of psilocin (Geiger et al., 2018). Not only psilocybin but also MAO inhibitors, such as harmine, were formed at the same time. This is relevant because psilocin is metabolised by MAO-A to the inactive derivative 4-hydroxyindol-3-yl-acetaldedyde (Blei et al., 2020). It has been speculated that for better protection against predators, some fungi produce both hallucinogen (e.g., psilocybin) and compounds that prolong hallucinogenic (e.g., harmine) effects because inactivation is impaired (Blei et al., 2020).

In Europe and the United States of America, several attempts were made in the 1960s to use psilocin in psychiatry. The Swiss pharmaceutical company Sandoz supplied for these studies psilocybin under the trade name Indocybin^®^. In such studies therapeutic applications of psilocybin were sought after. For instance, one asked whether psilocybin might be an appropriate tool to explore traits of personality or might help in understanding the mechanisms of a psychosis (Aldahaff, 1963; Charalampous et al., 1963; Leary et al., 1963). These studies were regarded as failures (review: Studerus et al., 2011) and psilocybin fell into disuse and was removed from the legitimate market. In recent years, a renaissance of psilocybin has occurred in terminally ill cancer patients and people suffering from depression (Ross et al., 2016). In these later studies, the effects of psilocybin on cardiovascular parameters in patients were reported. They noted tachycardia (Ross et al., 2016). However, the receptor mechanism has not been studied (Ross et al., 2016). There are scarce data from the older literature on the cardiac effects of psilocybin in animals. We found that both psilocin and psilocybin exerted a positive inotropic effect in isolated human atrial preparations (Dimov et al., 2023; Table 4). Hence, the proarrhythmic effects reported in clinical studies of psilocin and psilocybin might be due, in part, to their cyclic adenosine monophosphate (cAMP)-increasing effects on the heart.

The so-called “magic mushrooms” contain psilocin and its prodrug psilocybin; they are heat stable, meaning that they cannot be inactivated by heating extracts of the mushrooms. Psilocybin contains a phosphate at the phenolic part of the molecule, in contrast to its less polar metabolite, hallucinogenic psilocin (Figure 1). Therefore, psilocybin is more polar and thus soluble in water than psilocin, which requires organic solvents. Unexpectedly, we noted that psilocybin, usually regarded as an inactive precursor of psilocin, was active in isolated human atrial preparations to raise force of contraction (Dimov et al., 2023; Table 2). Hence, one may argue that the 5-HT_4_ serotonin receptor binding part of both compounds resides in the amino moiety of the drugs and not in the phenolic ring. However, this speculation needs to be confirmed by direct analysis of the crystal structure of psilocin and psilocybin bound to the recombinant human 5-HT_4_ serotonin receptor in the future.

The hallucinogenic effects of psilocin are usually explained by its agonistic potency (81 nM = Ki) at 5-HT_2A_ serotonin receptors, which is less than the potency of LSD at this receptor (Nichols, 2020). Moreover, psilocin binds to 5-HT_2C_ serotonin receptors (140 nM, Nichols, 2020). A complete list of the affinities of psilocin for 5-HT receptors was found in Geiger et al. (2018). From a cardiovascular point of view, the agonistic effect of psilocin on cardiac 5-HT_2A_ - and 5-HT_1_ serotonin receptors in the coronaries might cause harmful vasoconstriction. Stimulation of 5-HT_2_ serotonin receptors might lead to cuspid leaf defects. Binding to 5-HT_4_ serotonin receptors has never been reported (Geiger et al., 2018). It might be relevant that psilocin binds to H_1_ histamine receptors (Geiger et al., 2018). In the human heart, H_1_-histamine receptors induce bradycardia, have a negative dromotropic effect and might alter the force of contraction (review: Neumann et al., 2023c). This indicates a pleiotropic action of psilocin, possibly explaining its broad spectrum of effects on perception and awareness (Nichols, 2018).

Psilocybin undergoes a first-pass effect by metabolism in the liver by an alkaline phosphatase that can be inhibited by β-glycerolphosphate (Horita, 1963). 25% of perorally taken psilocin in rats is excreted unmetabolised (Kalberer et al., 1962). The fact that tropisetron antagonised the positive inotropic effect and positive chronotropic effect of psilocin and psilocybin is essential for two reasons (Dimov et al., 2023). This corroborates the conclusion that psilocin and psilocybin act via 5-HT_4_-serotonin receptors. Moreover, these findings suggest that one could treat magic-mushroom-intoxications with an approved drug, tropisetron. One could also use a more selective and potent 5-HT_4_ antagonist like piboserod which is however not readily available anymore (Kjekshus et al., 2009).

The potency of psilocin to increase the force of contraction could be increased by pretreatment of atrial preparations from 5-HT_4_-TG with a combination of the phosphodiesterase inhibitors cilostamide (1 µM) and rolipram (0.1 µM). This is consistent with our previous studies; cilostamide is a PDE III inhibitor, and rolipram is a PDE IV inhibitor (Neumann et al., 2019). We have previously used the concentrations of these drugs to potentiate the PIE of 5-HT in atrial preparations of 5-HT_4_-TG (Neumann et al., 2019). These findings support our conclusion that psilocin acts via the generation of cAMP. If the degradation of cAMP is reduced by reducing PDE activity, the agonist at the 5-HT_4_ serotonin receptor can lead to higher cAMP levels and, thus, higher force generation and elevated heart beating rate (compare Figure 1).

Extracts from the genus Psilocybe have been used at least as early as AD 300 in shamanic rites as hallucinogenic products in Middle America (Nichols, 2020). Psilocybe, however, is naturally occurring worldwide and, hence, has probably been used by people in many places (Nichols, 2020). Psilocin in mushrooms might have been used in Africa in the Sahara Desert, ancient Egypt and prehistoric caves in Spain (Geiger et al., 2018). In healthy volunteers, hallucinogenic doses (up to 30 mg per os) of psilocybin increased blood pressure (Hasler et al., 2004). For instance, 30 mg of psilocybin led to peak plasma levels of about 0.1 µM of psilocin and about 50% occupation of 5-HT_2A_ serotonin receptors in the brain, as measured by positron emission tomography (Madsen et al., 2019).

Psilocin and psilocybin could directly lead to tachycardia in users by stimulating 5-HT_4_ serotonin receptors in the sinus node. Tachycardia is a problem in patients with coronary heart disease because the oxygen supply of the heart might be reduced, and angina and myocardial infarction might occur. This tachycardia might be prevented or treated with tropisetron because tropisetron blocks (not only but also) 5-HT_4_-serotonin receptors. If one wants to treat depressive patients (there are currently 66 studies for psilocybin on file at clinical trials.gov, Table 2) with psilocybin, it might be useful to give an additional β-adrenoceptor antagonist to reduce the heart rate. Alternatively, one could prescribe, in addition to psilocin, a 5-HT_4_ antagonist that does not pass the blood–brain barrier (tropisetron easily passes the blood–brain barrier: Wolf, 2000). However, such drugs are currently regrettably not yet available.

Moreover, in normal dosing, one can question whether psilocin plasma levels are high enough to stimulate cardiac 5-HT_4_ serotonin receptors. As mentioned above, 0.1 μM of psilocin was measured under therapeutic conditions below any contractile effect. However, phosphodiesterase inhibitors (clinically used as levosimendan, milrinone, roflumilast, theophylline or caffeine) potentiate the contractile effects of 5-HT. We would argue that phosphodiesterase inhibitors would also potentiate the effects of psilocin. Finally, if depressive patients used an MAO inhibitor such as moclobemide, tranylcypromine, or deprenyl, the degradation of psilocin would be impaired, and higher plasma concentrations of psilocin might be reached; this could induce rapid heartbeat by simulating the cardiac 5-HT_4_ serotonin receptors. It has been reported that taking mushrooms led to cardiac death, probably via cardiac arrhythmia, in a patient 10 years after her heart transplant. The postmortal psilocin concentration in her plasma was 30 μg/L (0.15 µM, Lim et al., 2012; Table 3).

When giving increasing dosage of psilocybin to healthy volunteers, one did not notice even at the highest dosage (315 µg per kilogram body weight) changes in surface electrocardiograms or increased incidences of supraventricular or ventricular arrhythmias nor increases in heart rate (Hasler et al., 2004). However, at this dosing they noted an increase in blood pressure (Hasler et al., 2004). However, the study recruited only eight male and female volunteers with an age range of 22–44 years, so larger studies seem to be needed (Hasler et al., 2004). In a later clinical study on twelve healthy volunteers (gender and age were not reported), 0.6 mg per kilo Gram body weight was given (Dahmane et al., 2021). Under these conditions psilocybin, probably through its metabolite psilocin, increased the heart rate in these volunteers and tended to prolong the heart rate corrected QT interval. Hence, at high dosages psilocybin may cause detrimental torsade de pointes, a cardiac arrhythmia (Dahmane et al., 2021). The authors however, argued that the therapeutic dosing would be lower and therefore arrhythmias might not occur (Dahmane et al., 2021). In a third study, 32 volunteers were given 20 mg psilocybin through the mouth. The only cardiovascular alteration the authors reported was an increase in diastolic blood pressure (Ley et al., 2023). No other cardiovascular effects like arrhythmias were reported (Ley et al., 2023).

## Outlook

Hallucinogenic compounds are undergoing renewed interest in psychiatry. It remains to be seen how effective and safe they will be in the clinical routine treatment of psychiatric patients. Moreover, people will continue to take hallucinogenic drugs for thought-altering or recreational purposes. Hence, side effects remain a concern. This review provides a detailed oversight of the known cardiac effects in humans and how they can be predicted with some certainty, based on studies in experimental animals. One can summarize our review in the following way for inotropy in the human atrium: ergometrine is solely an agonist at H_2_-histamine receptors. Psilocin, psilocybin, DMT and 5-Me-DMT are solely agonists at 5-HT_4_-serotonin receptors. Finally, LSD is a dual agonist at H_2_-receptors and at 5-HT_4_-receptors. At least proarrhythmic side effects should be considered and treated using approved drugs that are antagonistic to the 5-HT_4_-serotonin or H_2_-histamine receptors. Controlled clinical trials should be initiated to make the therapeutic use of hallucinogenic drugs safer.
